# Axial Length Measurement Failure Rates with the IOLMaster and Lenstar LS 900 in Eyes with Cataract

**DOI:** 10.1371/journal.pone.0128929

**Published:** 2015-06-10

**Authors:** Colm McAlinden, Qinmei Wang, Konrad Pesudovs, Xin Yang, Fangjun Bao, Ayong Yu, Shishi Lin, Yifan Feng, Jinhai Huang

**Affiliations:** 1 School of Ophthalmology and Optometry, Wenzhou Medical University, Wenzhou, Zhejiang, China; 2 Key Laboratory of Vision Science, Ministry of Health P.R. China, Wenzhou, Zhejiang, China; 3 Flinders University, Adelaide, South Australia, Australia; Medical College of Soochow University, CHINA

## Abstract

**Purpose:**

To evaluate axial length (AL) measurement failure rate with the IOLMaster (Carl Zeiss AG, Germany) and Lenstar LS 900 (Haag-Streit AG, Switzerland) in eyes with cataract.

**Methods:**

Two hundred and ninety-six eyes of 170 patients with cataract were enrolled. Cataract type and severity were graded using the Lens Opacities Classification System III (LOCS III) and AL measurements were attempted with IOLMaster (version 5.4) and Lenstar LS 900 (version 1.1). Chi-squared analysis was used to assess if the difference in AL measurement acquisition rate was statistically significant between the two devices. The association of the different cataract types and severity with the AL measurement acquisition rate was evaluated with logistic regression analysis.

**Results:**

AL measurements were obtained in 184 eyes (62.16%) using the IOLMaster and 191 eyes (64.53%) using the Lenstar, which corresponds to a failure rate of 37.84% and 35.47% respectively. Chi-square analysis indicated no significant difference between the Lenstar and IOLMaster for AL measurement failure rate (x^2^ = 0.356, *P* = 0.550). Logistic regression analysis indicated no association between acquisition rates and cortical or nuclear cataracts with either device. There was a statistically significant association between acquisition rates and increasing severity of posterior subcapsular cataracts with the IOLMaster (β = -1.491, *P*<0.001) and Lenstar LS 900 (β = -1.507, *P*<0.001).

**Conclusion:**

The IOLMaster and Lenstar LS 900 have similar AL measurement failure rates (35–38%) for Chinese public hospital cataract patients. Increasing severity of posterior subcapsular cataracts was problematic for both devices.

## Introduction

Cataract surgery is one of the most commonly performed operations around the world.[1,2] Axial length (AL) measurement is a fundamental parameter in determining the most suitable intraocular lens (IOL) at the time of cataract surgery with inaccuracies in measurement resulting in a significant effect on the post-operative refractive result.[3,4] The IOLMaster (Carl Zeiss Meditec) which employs partial coherence interferometry (PCI), is a popular device used to measure AL. The device has been found to provide good precision in terms of repeatability and reproducibility for AL measurements with the additional advantages of being non-contact and user independent.[5,6] However, measurements are often difficult and inaccurate with corneal opacities, vitreous hemorrhages, macular diseases, advanced cataracts, vitrectomised eyes, staphyloma or poor fixation.[7–9]

The more recently developed Lenstar LS 900 biometer (Haag-Streit AG) is also a non-invasive and non-contact device but is based on optical low coherence reflectometry (OLCR).[10] It detects the reflected light wave from the surface of the anterior cornea to the retinal pigment epithelium. It is able to obtain nine parameters in a single measurement: corneal thickness, keratometry, white-to-white (WTW) distance, anterior chamber depth, pupillometry, lens thickness, retinal thickness at the point of fixation, AL, and visual axis decentration.[11] Few studies have assessed the performance of this device, particularly in terms of its performance in different types and severity of cataract.

The purpose of this study was to compare the AL measurement failure rate in patients with cataract with the IOLMaster and Lenstar LS 900. A further aim was to determine the association between the acquisition rate with the different types and severity of cataract.

## Methods

### Patients and examinations

In this prospective study, consecutive patients who were scheduled for cataract surgery at the Eye Hospital of Wenzhou Medical University, China, were invited to participate. The written Informed consent was completed after the nature of the study had been fully explained and research conformed to the Declaration of Helsinki. The research was approved by the Research Ethics Committee at Eye Hospital of Wenzhou Medical University. All patients underwent a full ophthalmic examination. Inclusion criteria included adult patients undergoing unilateral or bilateral cataract surgery. Exclusion criteria included severe corneal or vitreous opacities, severe retinal disease, significant systemic disease, or insufficient mental ability to comply with testing. Two hundred and ninety-six eyes of 170 patients (mean age 60.4 ± 6.5 years, range 51 to 80 years) were included in the study.

Each patient was examined on a slit-lamp biomicroscope by a single experienced examiner and the type and severity of cataract was recorded according to the Lens Opacities Classification System III (LOCS III) following pupil dilation with 0.5% tropicamide and 0.5% phenylephrine hydrochloride (Mydrin-P, Santen Pharmaceutical, Osaka, Japan). The LOCS III cataract severity was graded on a decimal scale for each of the four possible components: nuclear opalescence (NO), nuclear color (NC), cortical (C) and posterior subcapsular (PSC) cataract. For each cataract type or for nuclear color, higher scores indicate greater severity. The scale ranges from 0.1 (clear or colorless) to 5.9 (very opaque in cases of cortical and posterior subcapsular) or 6.9 (very opaque or brunescent in cases of opalescence and nuclear color). The AL measurement was attempted with the IOLMaster (Carl Zeiss Meditec AG, Jena, Germany, software version 5.4) and the Lenstar LS 900 (Haag-Streit AG, Koeniz, Switzerland, software version 1.1) in a random order. Patients were positioned with a chin and forehead rest, and instructed to fixate on the internal fixation target of each device. With the IOLMaster, a minimum of five consecutive measurements of AL were performed until five reliable readings where acquired with a signal to noise ratio (SNR) greater than 2.0 for each measurement. The composite-20 method was used with the IOLMaster as it has been shown to be the most successful at obtaining AL measurements compared with the standard, standard manipulated and composite-5 methods.[9] Similarly, with the Lenstar LS 900, at least three reliable measurements were acquired. The standard mode was used with the Lenstar LS 900 as the dense cataract measurement mode was unavailable with this version of the device. In unsuccessful cases with the automated mode, attempts were made manually with both devices.

### Statistical Analysis

The number of unobtainable readings with the IOLMaster and Lenstar LS 900 for each eye was recorded. Chi-squared analysis was used to compare the AL measurement failure rate between both devices. Eyes were then grouped into the various cataract types (NC, NO, C and PSC) and the number of unobtainable readings with each device was calculated and compared. Logistic regression analysis was used to investigate the association between the acquisition rate with the different types and severity of cataract.[12] All statistics were calculated using Statistical Package for the Social Sciences (SPSS, version 13.0, SPSS Inc.).

## Results

**Fig 1:**
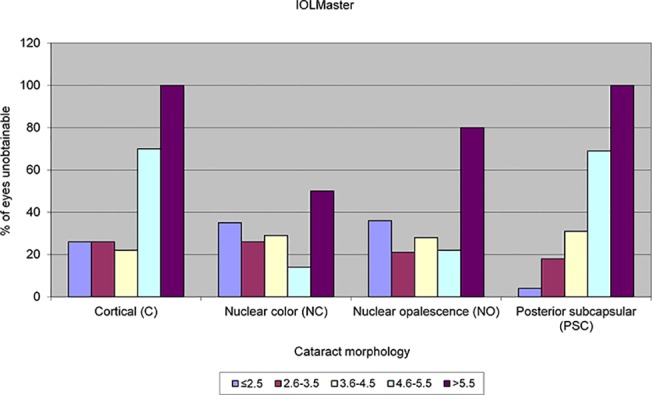
Failure rate in terms of percentage of eyes for the various cataract morphologies and LOCS III grading with the IOLMaster.

**Fig 2:**
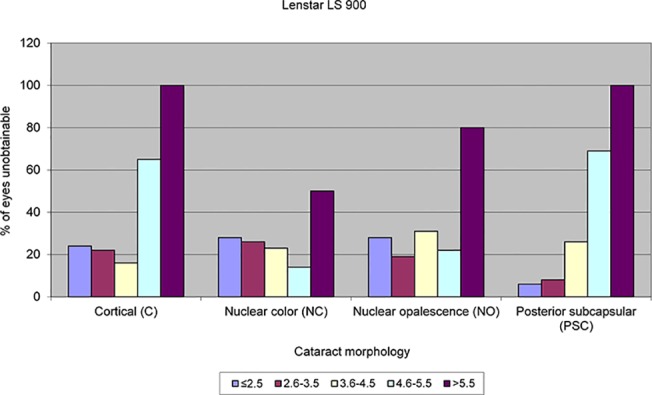
Failure rate in terms of percentage of eyes for the various cataract morphologies and LOCS III grading with the Lenstar LS 900.

LOCS III grading was not possible for 61 eyes due to advanced / mature cataract. The median and interquartile range for the LOCS III grading for the remaining 235 eyes is displayed in Tables 1 and 2. AL measurements were obtained in 184 eyes (62.16%) with the IOLMaster and 191 eyes (64.53%) with the Lenstar LS 900. The percentage of eyes unobtainable for the IOLMaster and Lenstar LS 900 for the various LOCS III cataract grades across the different cataract morphologies are displayed graphically in Figs 1 and 2 respectively. There was no significant difference in the total acquisition rates between the IOLMaster and Lenstar LS 900 (Chi-square X^2^ = 0.356, *P* = 0.550). The acquisition rates for the IOLMaster and Lenstar LS 900 for the different types and grades of cataract are shown in Tables 1 and 2 respectively. The general trend found was a lower acquisition rate with an increase in cataract severity. This was most evident with PSC cataracts and cortical cataracts but less so for nuclear cataracts. This trend was similar with both the IOLMaster and Lenstar LS 900.

**Table 1:** The number (percentage) of unobtainable axial length measurements with the IOLMaster for the different cataract types.

LOCS III	Cortical (C)		Nuclear color (NC)		Nuclear opalescence (NO)		Posterior subcapsular (PSC)	
	Number of eyes	Number (%) unobtainable	Number of eyes	Number (%) unobtainable	Number of eyes	Number (%) unobtainable	Number of eyes	Number (%) unobtainable
≤2.5	91	24 (26)	80	28 (35)	94	34 (36)	54	2 (4)
2.6–3.5	74	19 (26)	68	18 (26)	86	18 (21)	60	11 (18)
3.6–4.5	49	11 (22)	48	14 (29)	32	9 (28)	78	24 (31)
4.6–5.5	20	14 (70)	29	4 (14)	18	4 (22)	36	25 (69)
>5.5	1	1 (100)	10	5 (50)	5	4 (80)	7	7 (100)
Total	235		235		235		235	

Abbreviations: LOCS III = Lens Opacities Classification System III.

**Table 2:** The number (percentage) of unobtainable AL measurements with the Lenstar LS 900 for the different cataract types.

LOCS III	Cortical (C)		Nuclear color (NC)		Nuclear opalescence (NO)		Posterior subcapsular (PSC)	
	Number of eyes	Number (%) unobtainable	Number of eyes	Number (%) unobtainable	Number of eyes	Number (%) unobtainable	Number of eyes	Number (%) unobtainable
≤2.5	91	22 (24)	80	22 (28)	94	26 (28)	54	3 (6)
2.6–3.5	74	16 (22)	68	18 (26)	86	16 (19)	60	5 (8)
3.6–4.5	49	8 (16)	48	11 (23)	32	10 (31)	78	20 (26)
4.6–5.5	20	13 (65)	29	4 (14)	18	4 (22)	36	25 (69)
>5.5	1	1 (100)	10	5 (50)	5	4 (80)	7	7 (100)
Total	235		235		235		235	

Abbreviations: LOCS III = Lens Opacities Classification System III.

Logistic regression analysis was used to determine the association between cataract type (NC, NO, C and PSC) and severity with the acquisition rate (Table 3). A Bonferroni correction was applied (0.05/8), with a *P*-value for statistical significance set at 0.006. There was no association with NC, NO or C on the acquisition rate of AL measurements but there was a statistically significant association for PSC with both the IOLMaster (β = -1.491, *P*<0.001) and Lenstar LS 900 (β = -1.507, *P*<0.001). Increased severity of PSC was associated with a lower AL measurement acquisition rate.

**Table 3:** Logistic regression analysis assessing the association between cataract type and severity with acquisition rate.

Device		Cataract type			
		Nuclear color (NC)	Nuclear opalescence (NO)	Cortical (C)	Posterior subcapsular (PSC)
IOL-Master	β	-0.251	0.868	-0.433	-1.491
	*P*-value	0.387	0.013	0.011	<0.001*
Lenstar	β	-0.126	0.478	-0.323	-1.507
	*P*-value	0.670	0.167	0.061	<0.001*

*P<0.006.

Considering the unsuccessful cases (AL measurement failures), the following was also determined: Number of eyes unsuccessful with the IOLMaster but successful with the Lenstar LS 900: 13 eyes. Number of eyes unsuccessful with the Lenstar LS 900 but successful with the IOLMaster: 4 eyes. Number of eyes unsuccessful with both devices: 56 eyes. Of the patients undergoing bilateral cataract surgery, the number of eyes in which the AL measurement was unsuccessful with both devices and both eyes was 18 eyes (of 9 patients).

## Discussion

The purpose of the present study was to compare the IOLMaster (version 5.4) and Lenstar LS 900 (version 1.1) in terms of failure rates for acquiring AL measurements. The main benefits of this study include the large sample size 296 eyes of 170 patients and the study location of rural China with a significant proportion of patients with moderate to severe grades of cataracts. The study has also comprehensively documented the grade of cataract using the LOCS III decimal grading scale and has employed logistic regression analysis to determine the association between the acquisition rate with the different types and severity of cataract. Measurements of AL with the IOLMaster (version 5.4) and Lenstar LS 900 (version 1.1) were obtained in 62.16% and 64.53% of eyes respectively. This corresponds to a failure rate of 37.84% with the IOLMaster and 35.47% with the Lenstar LS 900. Despite the Lenstar LS 900 having a slightly lower failure rate than the IOLMaster, this difference was not statistically significant. Logistic regression analysis found that NO, NC or C cataracts had no influence on the failure of AL measurements with either the IOLMaster or Lenstar LS 900. However, there was a highly significant influence of PSC cataracts on AL measurements with both devices. Increasing severity of PSC was associated with a lower acquisition rate. In other words, more advanced or severe PSC cataracts result in a greater failure rate with both devices. Possible reasons for the high failure rate with PSC especially severe cases include poor fixation and as these opacities are closer to the nodal point of the lens, more light rays will be affected by this morphology of cataract compared to other cataract morphologies. Clinically PSC are well known to cause the most visual disability compared to other cataract morphologies, even in low grades of PSC. A previous study by Freeman and Pesudovs using an older version of the IOLMaster found a similar trend with PSC and increased failure rate.[8] They found that PSC was the principle reason for failure with a LOCS III PSC score of 3.5 defining the limit the IOLMaster was capable of measuring. In the present study, there were 78 eyes with a PSC LOCS III score between 3.6 and 4.5 and the IOLMaster failed to acquire a measurement in 24 of these eyes (30.77%). Interestingly, the Lenstar LS 900 failed to acquire a measurement in 20 of 78 eyes with a PSC LOS III score greater than 3.5 (25.64%). There were 36 eyes with a PSC score between 4.6 and 5.5 and both the IOLMaster and Lenstar LS 900 failed to acquire a measurement in 25 of these eyes (69.44%). There were 7 eyes with a PSC grade greater than 5.5 and both devices failed to acquire AL measurements in these 7 eyes. These results indicate that the newer version (version 5.4) of the IOLMaster has improved somewhat since the study by Freeman and Pesudovs in that it is able to acquire AL measurements in 65 of the 121 eyes with PSC grades greater than 3.5. This represents a failure rate of 36.47% in eyes with a PSC LOCS III score greater than 3.5. These findings mirror the findings of improved performance with version 5 of the IOLMaster in more advanced PSC cataracts reported by Hill et al.[9] There has been a further newer version produced by the company, the IOLMaster 500 and a recent study by Epitropoulos (n = 105 eyes) compared this IOLMaster 500 (version 7.1) to the Lenstar LS 900 (version not reported) finding no statistically significant difference in the success rate of acquiring AL measurements with the Lenstar LS 900 (standard mode) and composite-5 method (84.8% versus 83.8%, respectively; *P* = 0.782). However, the composite-20 method was statistically significantly more successful at acquiring AL measurements (92.4%) compared with the Lenstar LS 900 (*P* = 0.011).[13] This therefore suggests that the latest IOLMaster 500 (version 7.1) may have improved performance compared with version 5.4 of IOLMaster used in the present study as well as the Lenstar LS 900.

There were also measurement failures with lower levels of PSC cataract and part of this variability may be due to the LOCS III grading, in that it does not specify the location of the opacity. Two rays of light are used in the IOLMaster to measure AL and failure with lower levels of cataract may be caused by the opacity being located in a position within the lens which disrupts the path of one or both of these rays of light. Increasing severity of cortical cataracts tended to cause higher failure rates compared with nuclear cataracts, however, this finding was just outside the Bonferroni corrected statistical significance level (*P*<0.006). The majority of previous reports in the literature of IOLMaster performance report acquisition failure due to dense and PSC cataracts.[5,8,9,14–16]

The Lenstar LS 900 utilises the principle of OLCR and it is a more recent introduction to the market. A number of studies have been carried out to evaluate the quality of this new device, with most studies focusing on IOL calculation but few reporting failure rates.[17–29] Of the studies which have reported failure rates, AL measurement with the Lenstar LS 900 was unobtainable in 3% to 18% of eyes with or scheduled for cataract surgery, however these figures will be related to the severity of the cataract and hence variability is expected.[17,18,26,29–31]

A recent study by Hiu and Yi involving the IOLMaster (version 5) and the Lenstar LS 900 reported that from the 160 eyes, the IOLMaster failed to measure 28 eyes and the Lenstar LS 900 failed to measure 38 eyes. The measurement modes used with each device have not been reported.[32] Buckhurst and colleagues reported that the Lenstar LS 900 was unable to acquire measurements due to dense media opacities in a similar number of patients to the IOLMaster version 5 (9–10%) although formal cataract severity was not performed.[17] The present study indicates that the Lenstar performed similar to the IOLMaster. As found with the IOLMaster, increasing severity of PSC also proved problematic for the Lenstar LS 900 to acquire AL measurements (β = -1.507, *P*<0.001).

## Conclusion

The main clinical implications of this study are that failure rates remain high but comparable with both devices and posterior subcapsular cataracts continue to be problematic, especially with increasing severity of cataract. The IOLMaster version 5.4 has improved performance in dense and PSC cataracts than older versions but it still failed in some eyes. The failure rate was between 35–38% for both devices across all eyes. However, this figure depends on cataract severity and cannot be readily compared to other studies with differing levels of cataract. As can be seen from the results section, there is a wide spread of cataract severity across the different cataract types, with a significant number of eyes with advanced cataract. Advanced cataract is a common finding in the study location of Wenzhou in China. Similar failure rates with these devices may not been seen in other countries where less advanced cataract is seen with cataract surgery being performed at an earlier stage of the disease. A recent study involving the latest IOLMaster 500 (version 7.1) has reported improved performance compared with the standard Lenstar LS 900 measurement mode.
